# Meta-summaries effective for improving awareness and understanding of COVID-19 vaccine safety research

**DOI:** 10.1038/s41598-022-24607-6

**Published:** 2022-11-21

**Authors:** Spencer Williams, Joy Lee, Brett A. Halperin, Joshua M. Liao, Gary Hsieh, Katharina Reinecke

**Affiliations:** 1 Department of Human-Centered Design and Engineering, University of Washington, Seattle, USA; 2 Department of Medicine, University of Washington, Seattle, USA; 3 Paul G. Allen School of Computer Science & Engineering, University of Washington, Seattle, USA

**Keywords:** Public health, Human behaviour

## Abstract

Despite the efficacy, safety, and availability of COVID-19 vaccines, a lack of awareness and trust of vaccine safety research remains an important barrier to public health. The goal of this research was to design and test online meta-summaries—transparent, interactive summaries of the state of relevant studies—to improve people’s awareness and opinion of vaccine safety research. We used insights from a set of co-design interviews (n = 22) to develop meta-summaries to highlight metascientific information about vaccine safety research. An experiment with 863 unvaccinated participants showed that our meta-summaries increased participants’ perception of the amount, consistency, and direction of vaccine safety research relative to the U.S. Center for Disease Control (CDC) webpage, and that participants found them more trustworthy than the CDC page as well. They were also more likely to discuss it with others in the week following. We conclude that direct summaries of scientific research can be a useful communication tool for controversial scientific topics.

## Introduction

The COVID-19 pandemic has highlighted how low public trust and understanding of vaccine research has led to vaccine hesitancy^1^. In turn, hesitancy has contributed to suboptimal vaccination rates, which in turn contributes to an excess of preventable infections, hospitalizations, and deaths^2^. One of the most important causes of vaccine hesitancy is a lack of perceived safety^3–10^, in addition to low perceived efficacy^3,10^, logistic challenges^10,11^ and lack of trust in relevant institutions^4,5,9–12^.

Greater transparency and probity^1^, and communication of “facts over exhortations”^13^, are needed to improve the relationship between a vaccine-hesitant public and vaccine research. To meet this need and communicate much-needed vaccine information, this paper reports on online, interactive meta-summaries of COVID-19 vaccine safety research aimed at increasing public awareness, knowledge, and understanding in a transparent and trustworthy way.

Recent work has examined the potential of various online interventions to inform the public about vaccines. These online interventions for vaccine safety communication have shown some promise at informing the public through videos^14^, tailored information^15^, and interactions on social media^16^. They may focus on providing information for “fence-sitters” who seek to make informed decisions about vaccination^17^, so providing clear, high-quality, credible resources is key. These online interventions represent a scalable approach to increasing public awareness and understanding of vaccine safety information, and given how difficult it can be for even experts to stay informed about rapidly evolving research fields like those related to COVID-19^18^, this scalability is important. However, existing public health websites on COVID-19^19^, as well as other vaccine technology^20^, can be difficult to read, or fail to utilize data and graphics effectively, suggesting more work should be done to build online interventions grounded in the information and design needs of vaccine-hesitant groups.

Another approach to improving vaccine attitudes is to communicate the degree of scientific consensus among medical researchers^21^. While there is a large body of work showing that perceived scientific consensus can improve attitudes toward relevant scientific issues^21–23^, some have argued that these consensus messages may not be using an appropriate unit of analysis^24^. That is, these past consensus messages have focused on whether individual scientists agree with a particular finding (e.g., “vaccines are safe”); however, such an authoritative message may induce backfire effects^25^, and future consensus messages should highlight the *process* of consensus formation as agreement based on a given body of evidence. A more transparent approach may be more convincing, especially to the rising number of people who distrust scientific institutions in the U.S.^26^, as they are unlikely to defer to such appeals to authority.

To that end, we sought to develop ways of communicating COVID-19 vaccine safety research that highlights scientific consensus by summarizing direct evidence.

## Results

### Co-design interviews: qualitative results

We first present results from a set of co-design interviews on vaccine-hesitant people. Our primary research question driving these sessions was: What kind of meta-information about vaccine safety research is useful and relevant to vaccine-hesitant people, and how can we effectively communicate that information?

We recruited 22 U.S. adults to take part in our co-design interviews, identified from a screener survey we deployed on Amazon Mechanical Turk (MTurk). All participants indicated that they were either unvaccinated and currently hesitant to get vaccinated for COVID-19 (n = 10), or had previously been hesitant (n = 12). 11 were female, and 11 were male. 13 were White, 7 were Black, and 2 were Asian. For the full characteristics of our sample, see Table 1. Participant quotes are lightly edited for clarity.

**Table 1:** Participant demographics for co-design interviews.

ID	Gender	Race	Age	Education	Income	Political ideology
P1	Female	White	57	Bachelor's degree	Less than $10,000 USD	2. Somewhat conservative
P2	Female	White	31	Master's degree	$60,000–$74,999 USD	4. Somewhat liberal
P3	Female	Black or African American	33	Master's degree	$60,000–$74,999 USD	5. Very liberal
P4	Male	Black or African American	28	Bachelor's degree	Less than $10,000 USD	2. Somewhat conservative
P5	Female	White	38	Bachelor's degree	$100,000 USD or more	5. Very liberal
P6	Female	Black or African American	29	Bachelor's degree	$60,000–$74,999 USD	4. Somewhat liberal
P7	Male	White	31	Bachelor's degree	$75,000–$99,999 USD	5. Very liberal
P8	Female	White	42	Associate's degree	$25,000–$39,999 USD	2. Somewhat conservative
P9	Male	Asian	26	High school (or GED)	$25,000–$39,999 USD	3. Neither liberal nor conservative
P10	Male	White	37	Master's degree	$40,000–$59,999 USD	2. Somewhat conservative
P11	Male	Black or African American	29	High school (or GED)	$40,000–$59,999 USD	4. Somewhat liberal
P12	Female	White	55	Associate's degree	$40,000–$59,999 USD	4. Somewhat liberal
P13	Male	Black or African American	34	Bachelor's degree	$25,000–$39,999 USD	4. Somewhat liberal
P14	Female	White	33	High school (or GED)	$25,000–$39,999 USD	5. Very liberal
P15	Male	Black or African American	40	Master's degree	$100,000 USD or more	1. Very conservative
P16	Female	Asian	42	Bachelor's degree	$40,000–$59,999 USD	3. Neither liberal nor conservative
P17	Male	Black or African American	35	Associate's degree	$60,000–$74,999 USD	4. Somewhat liberal
P18	Female	White	48	Bachelor's degree	$40,000–$59,999 USD	2. Somewhat conservative
P19	Male	White	22	Associate's degree	$15,000–$24,999 USD	4. Somewhat liberal
P20	Male	White	38	High school (or GED)	$100,000 USD or more	4. Somewhat liberal
P21	Male	White	37	Bachelor's degree	$40,000–$59,999 USD	3. Neither liberal nor conservative
P22	Female	White	53	Bachelor's degree	$75,000–$99,999 USD	4. Somewhat liberal

Political ideology was measured on a 5-point scale (1 = Very conservative, 5 = Very liberal).

### Challenges using scientific papers

First, we found that scientific evidence was generally considered important, with several participants using research papers to help make decisions (“I had actually read a lot of scientific journals. I mean, I really dug deep into stuff like that.”—P22). However, these papers were typically not perceived as useful as information sources. First, they were difficult to access (“A lot of the times they're behind paywalls and it takes extra time to actually get the content”—P13). Second, even if they could be accessed, they were difficult to understand (“I didn't really read the raw science because it's way over my head.”—P20). Third, it can be difficult for non-experts to extract key takeaways, with multiple participants describing them as “boring” (“They're just kind of boring and I just want some basic information, like I don't really care to go in depth and that's what most of them are. I can understand what it's saying, but I just don't want to apply myself to do that.”).

However, our participants were interested in understanding the state of the research. Rather than searching through research papers, our prototypes that summarized key statistics and other information from the literature were considered useful. As P10 described:

This would help somebody like me, that's kind of on the verge of not knowing like, wanting to do what's best for everybody to help make a more informed decision without having to spend like 52 hours trying to source information, right? Going down the rabbit hole, and then being able to write it down or put it on a doc and okay, this says this and this, this, and it's like right here. You made the doc.

Given the need for a summary of relevant metascientific information in making vaccination decisions, we then analyzed what specific pieces of information participants considered useful, and how they would want it displayed.

### Quantity and consistency of research results

First, many of our participants were convinced by data showing how much research has been done on vaccine safety, and how consistently safe the COVID vaccines have been shown to be (“Something like 8.8 million people kind of makes me more comfortable to go take the vaccine.” -P16). Beyond the quantity, consistency was also important, with participants wanting to know results held over repeated studies (“I mean, if just one person does an experiment, that doesn't really prove much till it's like tested over and over again. So I think that part's really important.”—P14). Others had specific needs, such as who the participants of the studies were (“If I see that more severe side effects are happening more in women, I probably wouldn't get it.”—P8), depending on their specific questions.

### Indicators of trust

Not all participants were willing to trust that the data displayed was accurate. A common concern was that safety research (or how we were presenting it) was biased, so metascientific factors like funding source or institution were considered important (“let's just say Johnson and Johnson did the study, or that Moderna did the study. And I'm like, well, that's not unbiased.”—P10). This was considered the most important piece of information by several participants, specifically those who lacked trust in scientific institutions (“Funding source, it tells of a certain bias, I guess, for what they're looking for in their studies and whatnot, and who's funding them, who's giving them money, who made it possible. I think for me, that would be the most important for credibility.”—P9). Information on what country a study was conducted in was also considered relevant, for those who believed research done in the U.S. is untrustworthy (“I would look at studies from different countries, yeah.”—P1). Those who looked for this type of information expressed wanting to know the intentions behind the research; are researchers beholden to moneyed interests? Or are they doing research for altruistic reasons? Can they be trusted?

### Design considerations

In terms of design, participants were split on how they wanted to engage with the research. For some, having the ability to interact with and interrogate the data was useful, either to determine its credibility or its relevance to their own circumstances (“I like to have a lot of numbers. That way I can analyze it myself, figure things out. […] It's just the right amount of controllability. I can go from studies that have a hundred participants or studies that have a million, then hover over the icon and get even more of a breakdown of the information. So I think that's pretty amazing.”—P17). Others had no interest in this level of interrogation, preferring a clear message explaining key takeaways (“Interaction feels like a waste of time, especially when it's a message that could still be conveyed with a simple, still graphic that's already sort of developed for me. I just want a clear, clean, simple presentation of information that I can really just digest at a glance.”—P20).

This difference also emerged explicitly in many of our participants’ sketches. While there was some variety in form and function, most commonly participants generated two-tiered, interactive infographics. They often began with high-level, plain-language, bulleted summaries of the state of the research, to ensure the key takeaways are clear. This was usually supplemented by an interactive graph or other visualization, providing additional details on demand for those who want to more deeply interrogate the research. These often involved hovering/clicking on studies to propagate details like funding source, sample size, study population, etc. This general approach allowed for easy interpretation from participants who had no interest in exploring the details of the literature (“Hey, I'm just asking this one question. I just want one sentence really. And then if I'm interested by the answer, I'll click read more. Cause otherwise…this is just too much or overwhelming and I'll just close out of it.”—P19). For others, however, providing transparent details on the metascientific information (institutions, authors, funding sources) could improve trust, and help users make more nuanced decisions about the literature.

Finally, some participants were concerned about bias in the system. For some, the explicit narrative provided by some of our prototypes (e.g. one providing a text-based summary) could feel it’s leading readers to a specific conclusion, and glossing over conflicting information, as P4 describes:

I feel like there would be some sort of study that maybe majority might lead to like this direction, but I'm sure there is some research out there, maybe like a very minute number, that might have opposite results than what's presented here. I would say having that as well might make the reader feel that they're the ones making the decision versus, you know, leading them to a certain decision.

To address this, visualizations that clearly display the full range of studies on vaccine safety could be considered more transparent, and less biased (“The bias was really just coming from how the information is structured or worded. […] But with this [a scatter plot graphing all studies in our sample] you can look at each thing one at a time, so it doesn't feel biased anymore.”—P19). Such an approach would likely rely on users’ trust that the displayed range of studies is representative of the full literature.

Based on the findings from these co-design sessions, we generated a list of design requirements for scientific meta-summaries in this domain:

1. Provide simple, concise, text-based summaries of the information.
2. Provide interactions for details-on-demand, to provide deeper insights for those who want to interrogate the literature and ensure credibility.
3. Visually convey the quantity of research.
4. Visually convey the consistency of research.
5. Provide key metascientific signals of credibility (e.g. funding source).
6. Signal that the research displayed is representative of the full body of COVID-19 vaccine safety research.

## A meta-summary of COVID-19 vaccine safety research

**Figure 1:**
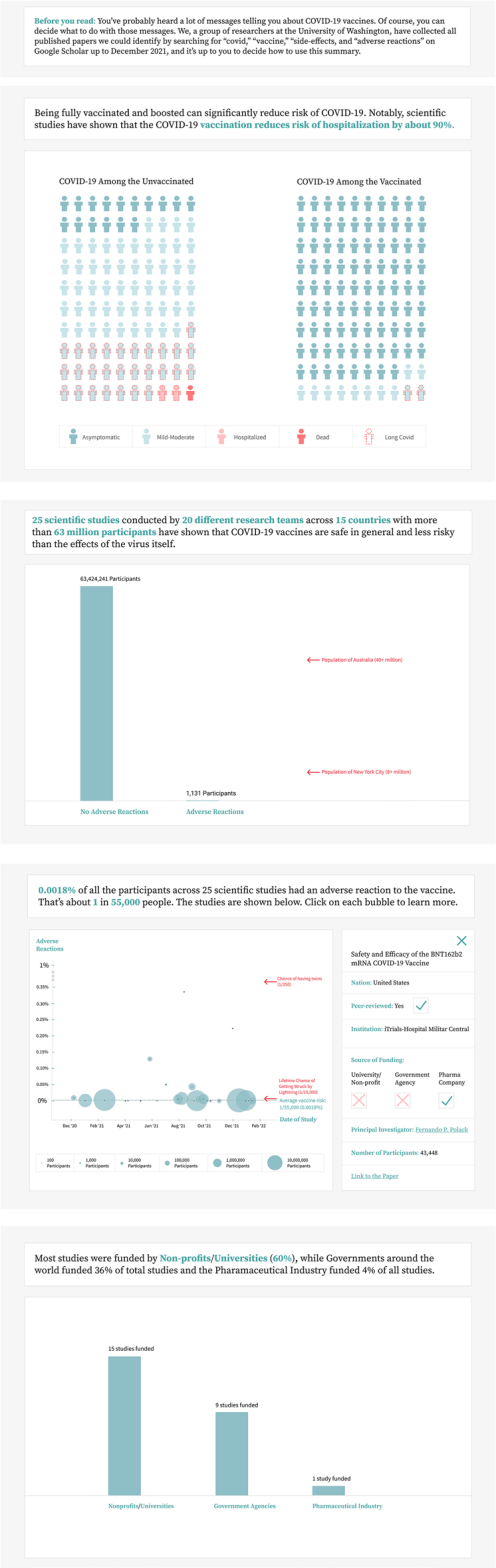
A screenshot of the full version of our intervention. The top visualization is an icon array representing the efficacy of mRNA vaccines at preventing serious COVID-19 outcomes (only present in the full version). Next is a bar chart depicting the number of participants or individuals across studies with or without serious adverse reactions to the vaccine. Below that is an interactive scatterplot depicting the number, size, and risk estimates of studies over time, with the right pane providing additional details. At the bottom is a bar chart representing the most common sources of funding across studies.

Based on the above insights and following our design requirements, we designed an interactive meta-summary meant to provide metascientific information our participants considered valuable when assessing vaccine safety research, in a format that would be useful to them. Following the formatting conventions participants used (high-level takeaways, details on demand), we designed four visualizations describing key pieces of information based on the needs we identified (see Fig. 1).

We developed and tested two versions of our intervention. In general, our co-design participants were concerned about safety, they considered a large amount of consistent research to be useful in assessing safety, and they considered funding source to be useful in determining credibility. Our interventions prioritized signaling this information. In the first version (“safety only”), we first included visualizations about the number of participants across studies in our sample, using tick marks [e.g. “Population of Australia (40 + million)”] to contextualize the combined size of the studies in our sample. We next included study estimates of vaccine safety risk over time in a scatter plot (highlighting the consistent low risk estimates), where participants could click each study for additional details (providing a transparent overview of the research space). We specifically used serious adverse events as outcomes, defined by the studies on COVID vaccines in our sample, which included outcomes like myocarditis, anaphylaxis, and GBS. Finally, we included a graph of the frequency of different funding sources, which highlighted that most studies were funded by their own university or non-profit organizations.

In the second version (“full version”), we also included a graph depicting the efficacy of the mRNA vaccines at preventing serious outcomes, in order to additionally address the belief shared by a subset of our participants that the vaccine was ineffective or unnecessary.

For both versions, we also drew on qualitative work that suggests providing “facts over exhortations” for vaccine-hesitant people^13^. This is in-line with research on psychological reactance^30^, whereby people respond negatively when they perceive that their freedom is being threatened. To address this, we adapted an “inoculation” message from past work^31^ to remind participants that they are free to use the information however they wish:

You’ve probably heard a lot of messages telling you about COVID-19 vaccines. Of course, you can decide what to do with those messages. We, a group of researchers at the University of Washington, have collected all published papers we could identify by searching for “covid,” “vaccine,” “side-effects, and “adverse reactions” on Google Scholar up to December 2021, and it’s up to you to decide how to use this summary.

## Experimental evaluation

To test whether our interventions improved vaccine hesitant people’s awareness, opinion, and trust of vaccine safety research, and their intention to get vaccinated, we conducted a pre-registered (https://aspredicted.org/blind.php?x=56W_KT2) experiment on MTurk. There were three conditions: the safety only version, the full version, and a baseline condition where we included information from the U.S. Center for Disease Control’s (CDC) page on vaccine safety data.

In total, we received 2198 responses after systematically removing responses which did not meet appropriate checks (see “Methods” for details), and analyzed the responses of the 863 of those who were unvaccinated.

Compared to the CDC condition, we found that participants were more likely to agree that there was more research than they thought (F(2, 871) = 5.15, p = 0.006, η^2^ = 0.012), more consensus than they thought (F(2, 871) = 9.65, p < 0.001, η^2^ = 0.022), and that the research showed they were safer (F(2, 871) = 15.31, p < 0.001, η^2^ = 0.034) than they thought before viewing our interventions. Moreover, participants rated our intervention as more trustworthy than the information on the CDC page, F(2, 871) = 3.36, p = 0.035, η^2^ = 0.008. See Table 2 for pairwise comparisons.

**Table 2:** Pairwise comparisons for significant effects.

Comparison	Cohen’s d	95% CI	p (Dunnett’s test)
**Research amount**			
Safety only—base	0.22	[0.07, 0.37]	0.004
Full version—base	0.17	[0.02, 0.32]	0.058
**Research consensus**			
Safety only—base	0.28	[0.13, 0.43]	0.006
Full version—base	0.21	[0.6, 0.36]	< 0.001
**Research safety estimate**			
Safety only—base	0.36	[0.21, 0.51]	< 0.001
Full version—base	0.23	[0.08, 0.38]	< 0.001
**Trust in information**			
Safety only—base	0.19	[0.04, 0.34]	0.030
Full version—base	0.28	[0.13, 0.43]	0.087

However, there was no significant effect of our intervention on participants’ concerns about vaccine safety, F(2, 871) = 0.89, p = 0.413, η^2^ = 0.002. There was also no effect on their perceived credibility of vaccine science overall, F(2, 871) 1.36, p = 0.256, η^2^ = 0.002. Finally, we ran a logistic regression testing whether participants increased their intention to get vaccinated, but there was again no significant effect (see Table 3).

**Table 3:** Logistic regression of intervention condition on increased intention to get vaccinated.

Variable	β	SE	p	OR
Intercept	− 2.55	0.22	< 0.001	
Safety only	0.20	0.30	0.517	1.22
Full version	− 0.02	0.32	0.945	0.98

### Longitudinal effects

We then conducted a follow-up survey with participants from our experiment between 1–2 weeks after the initial deployment (N = 547), where we asked whether they had thought about the intervention, discussed it with others, been inspired to do more research on COVID-19 vaccines, or made a decision about whether to get vaccinated.

We found that participants who viewed our intervention were more likely to have discussed it with others than those who viewed the CDC version (see Table 4). There were no significant effects on other longitudinal activities.

**Table 4:** Logistic regression of intervention condition on whether participants spoke with others about the intervention.

Variable	β	SE	p	OR
Intercept	− 1.28	0.17	< 0.001	
Safety only	0.51	0.24	0.031	1.67
Full version	0.40	0.24	0.098	1.49

### Qualitative results

To help explain our results, we asked participants in both the initial experiment and the follow-up to answer qualitative questions about their initial impressions of the intervention, and the circumstances around any thoughts/discussion/decisions they had in the follow-up.

First, in-line with previous qualitative results, with the amount and consistency of research, and funding sources, were considered useful signals:

First, I didn't realize there had been so many studies on the vaccine, and the correlations of those results were encouraging. I was also encouraged to know that the majority of the research studies were not funded by a government ensuring the science speaks for itself. I would say overall the information slightly improved my feelings towards the safety of the vaccine.

However, there were also a number of reasons the intervention did not work for other participants. First, a number of participants had little trust for science as an institution, believing that researchers may be biased (“I simply don't trust universities either. They are too biased in everything and if anyone even came out with research that shows there's something wrong, they would be canceled and I think that motivates everyone to walk in lockstep with the mainstream narrative.”). This may tie in with the belief that vaccine researchers are financially motivated to publish only positive results (“This information did not change my opinions about vaccine research as I do not believe it is reliable or accurate as researchers are highly motivated to portray the vaccine positively due to enormous financial incentives and funds.”).

Second, there were lingering concerns about potential long-term effects, which participants felt this body of research could not yet address (“It made me feel a bit more confident about it but we still don't know long term effects.”).

Third, as our interventions focused on safety, this was not the main concern for all participants (“I believe that the COVID jabs are "safe", so your research didn't speak to my issue. I haven't gotten one because they are NOT effective at preventing transmission.”). Even participants in our full version were not convinced of the importance of the vaccines (“I still do not believe it to be safe or effective.”). The above factors, as well as a general lack of trust in pro-vaccine messages (“Y'all are liars and I laugh at your dumb propaganda.”), represent additional obstacles for vaccine safety communication.

For the follow-up survey, in-line with our finding that participants who saw our intervention were more likely to discuss it than the CDC page, we found having clear, transparent numbers may help empower those who have already been vaccinated to have discussions with friends who are still hesitant (“I have a relative who was […] spouting nonsense. The information I was shown on the survey gave me a few actual facts rather than just calling him an idiot.”).

## Discussion

In this work, we showed that meta-summaries of a scientific topic can serve as effective communication tools. Our intervention was successful at informing vaccine-hesitant people about the amount, stability, and valence of COVID-19 vaccine safety research. Importantly, it was considered more trustworthy than information from the CDC website, an important communication platform during the pandemic. We have also shown that an approach using more direct scientific evidence, rather than statements of expert consensus, can be effective at establishing scientific consensus on a controversial topic^24^.

While our research showed that scientific meta-summaries can provide trustworthy information, our intervention did not significantly increase people’s intention to get vaccinated, suggesting a need for future work. One issue was that our intervention did not affect people’s perceived credibility of the underlying science; even if they believe the information was reported accurately and honestly, they may still have a broad distrust of the scientific establishment.

One possible approach to increasing credibility of science as a whole would be to empower audiences to better navigate and interrogate vaccine research. Beyond increasing trustworthiness via a show of vulnerability to critical assessment, feelings of powerlessness have been associated with susceptibility to conspiracy theories^32,33^, suggesting that interventions to empower people can potentially work to reduce conspiracy-related beliefs^34^. By guiding skeptical audiences to critically-but-competently evaluate the studies provided, or on how to use the metascientific information we provide as useful heuristics (e.g. for research quantity or credibility), it may be possible to further increase trust and understanding of science using a metascience-based approach as we have.

Finally, although safety concerns have been a major issue for vaccine rejection, they are not the only ones; concerns about efficacy, logistics (e.g. cost, transportation), and trust are also important considerations^3–5,9–12^. Thus, even interventions that affect participants’ assessment of safety research, while important, may not be adequate in convincing those participants to get vaccinated, without a more holistic effect on other key concerns.

## Methods

Data collection for our screener survey, co-design, and experiments had IRB approval by the University of Washington Human Subjects Division. All procedures were carried out in accordance with relevant guidelines, and informed consent was obtained where applicable.

This paper reports on a set of online meta-summaries—text and visualizations summarizing high-level information about the state of the research domain—about COVID-19 vaccine safety research, developed using a human-centered design (HCD) process^27^. We first conducted qualitative interviews with a co-design component^28^ with vaccine-hesitant people in the U.S, in order to learn why they were hesitant to get vaccinated and to have them sketch out ways of accessing scientific knowledge about vaccines. As a method, co-design can help build empathy between researchers/designers and participants/users^29^, an important consideration when eliciting feedback from people hesitant about the COVID-19 vaccines^13^. It also allows us to frame the interviews around participants’ needs, with the intention of establishing buy-in from participants who may not otherwise trust research on Covid-related topics.

### Co-design interviews

Co-design is a research method where participants are asked to engage directly in the design process^28^. In our sessions, we asked them to sketch designs for a research summary that would best meet their needs. By framing interview sessions as a way of eliciting feedback to produce tools for our participants, we sought to establish a co-operative relationship and build empathy^29^. We expected this would help participants be more candid with their opinions and needs, particularly when working with a population with lower-than-average trust of science^4,5,11^.

To recruit participants, we deployed a screener survey on MTurk (n = 676), where we asked “Are you—or at some point were you—hesitant to get the COVID-19 vaccine (for example, Pfizer, Moderna, Johnson & Johnson)?”, “If you could ask any question about the COVID-19 vaccines (for example, Pfizer, Moderna, Johnson & Johnson), what would you ask?”, and demographic questions. We emailed those who responded that they were either currently hesitant, or at some point were hesitant, to get the vaccine, and indicated their willingness to participate in an interview. In total we reached out to 153 participants for a final sample of 22 who agreed to participate (14.4%).

Our full interview protocol is provided in Appendix A of the Supplementary Materials. We began by asking preliminary questions about where participants get information about COVID-19 and vaccines, and what made them hesitant. We then provided a list of information about scientific research (information about studies, their findings, their authors, institutions, funding sources, participants, etc.), and asked what they might find useful when making vaccination decisions. We then showed off three mockups: a text-based summary of relevant literature, an interactive scatter plot, and a tool to simulate and visualize possible outcomes of getting vaccinated. We asked what was (not) useful about these mockups, and how they could be improved. We then asked how these might be implemented into social media or search engines, before ending by asking them to sketch a version of the tool that would be best suited to their needs. Sessions were approximately 45–60 min.

Interview data was analyzed using a thematic analysis approach^35^. We first transcribed the audio for each interview, open coded the first five to develop a coding scheme, then close coded the full set. Each coded section from each interview was added to a single document under that code, where we extracted high-level summaries of each code. These were then combined to develop a number of themes, with particular focus on the information needs and goals of different participants.

### Experiment

#### Distribution

To test our interventions, we deployed an experimental survey on MTurk. Participants were only allowed to take the survey if they were on a laptop or desktop computer, with mobile users being automatically removed, to ensure our prototypes displayed correctly. It was posted to MTurk with the title “Read about Covid-19 vaccines and answer a survey [CANNOT USE MOBILE]”, the description “Read some information about Covid-19 vaccine safety research and give us your opinions. [Mobile responses not allowed; must use laptop or desktop computer]”, and was tagged with the keywords “survey”, “research”, “vaccine”, and “covid”.

#### Conditions

**Figure 2:**
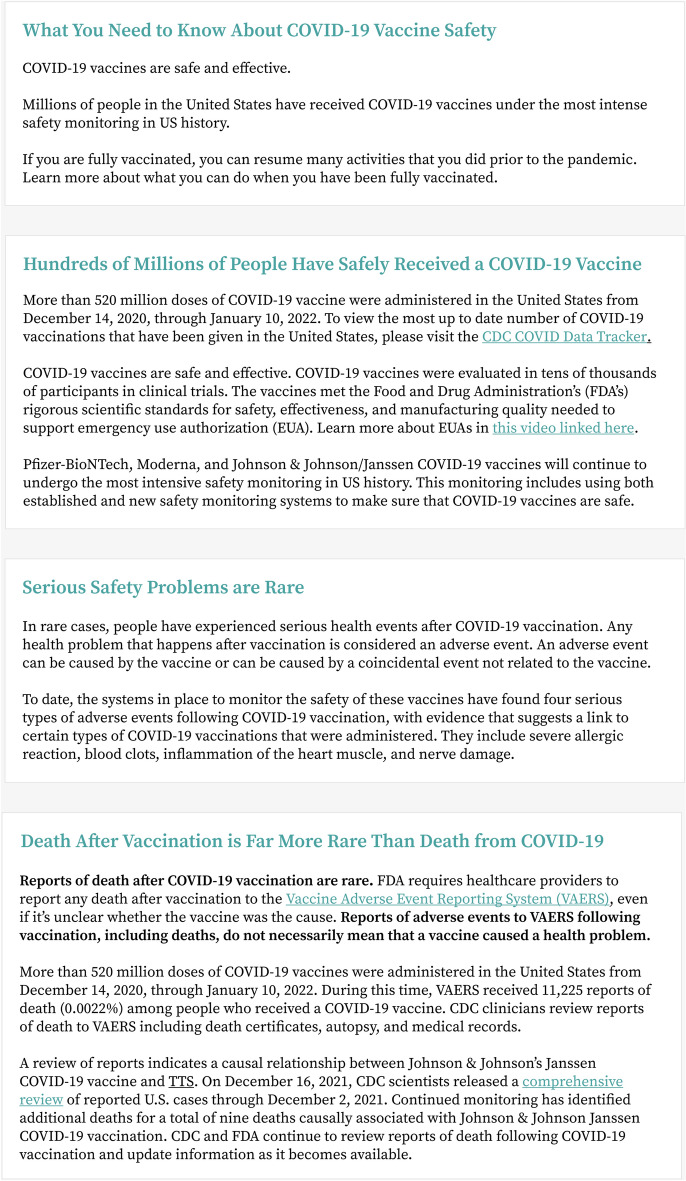
A screenshot of the condition we developed by pulling information from the CDC page on COVID vaccine safety research.

Participants were randomly assigned into one of three conditions: the full version, the safety only version, and a baseline using information from the then-currrent CDC page on vaccine safety (There were three conditions: the safety only version, the full version, and a baseline condition where we included information from the CDC’s page on vaccine safety data (see Fig. 2). We chose this as a baseline because, like our interventions, it serves as a high-level overview of safety data on COVID-19 vaccines. It mirrors our intervention in that it provides the amount of research (“More than 520 million doses…”) and safety estimate (“VAERS received 11,225 reports of death (0.0022%)…” Thus, it represents a then-current standard in how this information is being communicated to a U.S. audience by an official government body. Any improvement we show relative to this baseline represents an improvement on current communication with similar goals.

#### Measures

We measured vaccination status by asking “Have you been vaccinated for COVID-19?” and for those who responded “yes”, “Have you received a COVID-19 vaccine booster?” Vaccine intention was measured by asking participants “How would you describe your intention to get [next dose]?” (definitely/not sure/definitely not). Participants were also asked to their vaccine hesitancy concerns on a 5-point likert scale (“I am not worried about getting infected with COVID-19”, “I do not believe the COVID-19 vaccines are effective”, “I do not believe the COVID-19 vaccines are safe”, “It is too costly to get a vaccine (for example, can’t get transportation, can’t take time off work)”).

We measured credibility of US vaccine research using three subdomains from prior work: trustworthiness, competence, and benevolence^36^. Specifically, they were asked to rate their agreement with the following statements on a 5-point scale: “I think COVID-19 vaccine safety research in the United States [is trustworthy/is competent/protects public interest]." We also measured their trust of the information in the meta-summary itself, using agreement with two statements from prior work (“I think the information I just read was [believable/balanced]" on a 5-point scale^37^.

We measured their agreement with statements about research amount (“There is more research on COVID-19 vaccine safety than I thought there was before taking this survey.”), research consensus ("Research on the safety of COVID-19 vaccines is more consistent than I thought before taking this survey."), and direction of the research ("Research on the safety of COVID-19 vaccines shows a lower rate of adverse reactions than I thought before taking this survey.") on a 5-point scale.

#### Procedure

Participants began by reading a brief explanation of the experiment, including details of what they will be asked to do. Participants were first asked for their vaccination status, vaccine intention, and vaccine hesitancy concerns. Next, they were asked to rate the credibility of US-based vaccine research.

Participants were then randomly assigned to one of our three conditions. They were instructed that they will “see a set of information about the safety of COVID-19 vaccines. Please read through the information on the page before moving on with the survey.” After they left the page, they were given an optional text-entry question asking “How (if at all) did the information you just saw affect your opinions about covid vaccine research?”.

After interacting with the meta-summary, they were asked about their trust of the information on the page. Next, they were asked the same set of questions about vaccine research credibility, as a pre-post measure. After that, they were asked about the amount, consistency, and direction of vaccine research, before responding to the same questions about vaccine intention and reasons for hesitancy as above, again as a pre-post measure.

Finally, participants were given the short need for cognition (NFC) scale^38^, and graph literacy scale^39^, before completing a set of demographic questions and ending the survey.

#### Analysis

For analysis and recruitment, we followed the plan laid out in our pre-registration, with three changes. First, when analyzing intention to get vaccinated, we used a logistic regression instead of an ordinal model due to a failure of the latter to converge as specified. Second, we oversampled to ensure that we had an adequate number of *unvaccinated* participants, as our original sampling plan did not take this into account. Finally, we noticed in the qualitative responses that several participants had seemingly copy-pasted sections of Covid-related articles found online. Many of these were identical to each other, and we concluded these were likely either bots, or the same participants using identical responses across IP addresses.

To improve data quality, we decided to remove responses with this type of qualitative answer. First, to ensure we were removing copy-pastes and not just low-effort responses (e.g. “good”, “no”), we first set a minimum character limit of 50 for qualitative responses. Next, we compared each response against all others, until identifying one with a Levenshtein distance of less than 10% the length of the response. This helped identify obvious copy-pastes with minor errors, like the following:

- “THE AVAILABLITY OF A SAFE AND EFFECTIVE VACCINE FOR COVID-19”
- “THE AAVAILABILITY OF A SAFE AND EFFICTIVE VACCINE FOR COVID-19”

This procedure identified 268 likely bots out of 2393 total unique IDs.

For analysis, although our pre-registration specified we would analyze specific vaccine hesitant sub-groups, we did not observe any differences or interactions between these groups, and chose not to discuss them here due to a lack of theoretical relevance in interpreting our results.

#### Follow-up survey

Finally, our follow-up survey was not pre-registered, and was developed within the week following our initial survey deployment. It was deployed on MTurk, was made available only to those who had completed our initial experiment, and ran 8–14 days after that experiment. The survey on MTurk was titled “Follow-up survey to "Read about Covid-19 vaccines and answer a survey [March 8–9]", included the description “This is a follow-up to a survey we launched during March 8–9. You will be asked a few questions about what you remember from the vaccine safety research summary you were shown, and how it has affected you.”, and was tagged with the keywords “survey”, “covid”, “vaccine”, “follow-up”.

We first showed them a screenshot of our different conditions to remind them which survey we were referring to. Next, we asked them to indicate which types of activities (“Thought about the research summary from the original survey,” “Mentioned the research summary (or information from it) in a conversation with someone else”, “Searched for research about COVID-19 vaccine safety,” or “Made a decision about whether or not to get vaccinated”) they had engaged in since participating in the original experiment (all on 5-point scales). For each option they indicated, participants were asked to describe in more detail via open text entry. Finally they were asked to rate how much they agreed with the statement “Reading the research summary on COVID-19 vaccine safety helped me feel more confident talking about COVID-19 vaccine safety with others,” whether they had any additional thoughts, and were compensated.

## Supplementary Information

Supplementary Information.

## Data Availability

Anonymized interview transcripts and experimental data will be made freely available to any researcher wishing to use them for non-commercial purposes. Please contact the corresponding author for access.
