# Author Correction: Meta-summaries effective for improving awareness and understanding of COVID-19 vaccine safety research

**DOI:** 10.1038/s41598-022-27366-6

**Published:** 2023-01-03

**Authors:** Spencer Williams, Joy Lee, Brett A. Halperin, Joshua M. Liao, Gary Hsieh, Katharina Reinecke

**Affiliations:** 1 Department of Human-Centered Design and Engineering, University of Washington, Seattle, USA; 2 Department of Medicine, University of Washington, Seattle, USA; 3 Paul G. Allen School of Computer Science & Engineering, University of Washington, Seattle, USA

Correction to: *Scientific Reports* https://doi.org/10.1038/s41598-022-24607-6, published online 21 November 2022

The original version of this Article contained an error in the name of the author Brett A. Halperin which was incorrectly given as Brett Halperin.

The original Article has been corrected.

