# Supplementary material for: Meta-summaries effective for improving awareness and understanding of COVID-19 vaccine safety research
Source: Sci Rep. 2022 Nov 21;12:19987. doi: 10.1038/s41598-022-24607-6 (PMC9676710; doi:10.1038/s41598-022-24607-6)
Supplement: Supplementary file 1 — Supplementary Information. [file 41598_2022_24607_MOESM1_ESM.pdf]

Co-Design Workshop Facilitator Agenda

FAQ for participants: https://www.cdc.gov/coronavirus/2019-ncov/vaccines/faq.html

(2 mins) **Facilitator introduction**

(3 mins) **Introduction.**

Hello! Thank you so much for agreeing to participate in this interview. Basically, our goal here is to design a way of summarizing research results regarding COVID-19 vaccines for people in the most useful way possible. We're interested in hearing about a variety of viewpoints, since we want to understand factors around trust and confidence in COVID-19 vaccines

Remember, your participation today is totally voluntary, and if at any point you feel uncomfortable during this session, you can always let me know, skip certain questions, or leave the call entirely. Also, although we may use quotes from this session in a research paper we write about this study, they will be completely anonymized, and you can let us know if you want anything you say here be cut from the record so we don't report on it. We will not release any audio or video from this session.

Pause for questions

Do we have your permission to record this session?

Ground rules:
Facilitator cameras on and want you all to be comfortable on the call so
Participant camera/no camera? (up to participant)
Open discussion – no judgement space, really just trying to gather your perspective
Always ok to skip questions or leave the call

Pause for questions

House keeping:
Mute button – use when not talking
Camera

Pause for questions

(20mins) **Preliminary Questions (Q1-2 and Q4-5 Information/source focused. Q3 Hesitance focused)**

1. First, I want to ask you some questions about where you've gotten your information about the COVID vaccines. Could you explain where you usually get your information?

    a. Followup: Have you ever seen information about it on social media?
    b. Where was that information coming from? For example, news articles, scientific papers?
2. If you were hesitant at any point to get the COVID vaccines, could you explain what made you hesitant?
3. Have you ever read news articles or scientific papers about COVID vaccines?
    a. Probe 1: Did you intentionally look for the information? What motivated you to access the news article or scientific paper? (did you have a specific question?)
    b. Probe 2: If not, how did you come across it?
    c. Probe 3: Did you trust it or not? Why or why not?
    d. Probe 4: Who wrote it? Was it difficult or easy to understand? Do you have any questions about the article?
4. Do you currently have or have you ever had any questions about COVID vaccines or vaccine research that you wished you had answers to, but couldn't find anywhere?
    a. [Write down answers so that you can later use them to point out how they are answered in the mockups or not]

(25mins) **Feedback about Mockup**

Now, I'll show you several mockups of a tool we're planning to build to summarize existing research papers about vaccines. Remember, we're still working on the design and haven't actually built any of it yet, so be completely honest with your feedback.
I should also mention that, even though this is just a mockup, the data we're displaying with it is real, so keep that in mind.

[Show off the mockup(s)]

[Prompt for variables]
Which of these are most relevant to you?
Is there anything missing here that you'd like to know about vaccine research?

[Prompts for each mockup]
Now, I've got a few questions that we can discuss together:

1. "How might this tool help you make decisions about the vaccine?"
2. "What information is missing that could help you make an informed decision?"
3. Is there something about this information/tool that you find misleading or off-putting?

[Additional prompts per mockup, optional]
1. Executive summary
    a. NA
2. Scatterplot

a. Are there any other comparisons that might be useful (other vaccines, other medical interventions)?
b. What other information about individual studies would be helpful when deciding whether to trust them?

3. Simulator
    a. What information would you like about where these simulations are coming from?

[For interaction mockups]
Now, I'm going to show you some different ways we might show you this tool.

[Prompts for each mockup]

1. In what ways would this interaction be useful?
2. In what ways would this be unhelpful?

Sketching Task

For this last activity, I'm going to ask you to sketch out ideas for a tool. You can use this online sketchpad if you're on a computer, a physical pen and paper, or anything you have access to. If not, you can also just spend some time thinking about what it might look like, and describe it in words.

I'll give you about 5-6 minutes to roughly sketch something out, and then we can discuss what you came up with.

1. "Could you explain what you came up with?"
2. "How could we integrate this into the social media that you usually use?"

Wrap-Up

1. Before we wrap up, do you have anything you want to say that you didn't get a chance to during our session?
2. Do you have any last questions for me?
3. Thank you! [remind how will get your incentive]
